# Supplementary material for: Association of intraabdominal fat with the risk of incident chronic kidney disease according to body mass index among Korean adults
Source: PLoS One. 2023 Feb 9;18(2):e0280766. doi: 10.1371/journal.pone.0280766 (PMC9910748; doi:10.1371/journal.pone.0280766)
Supplement: S1 File — (DOCX) [file pone.0280766.s001.docx]

**Supplements**

Supplementary Figure 1. Flow chart of enrolled participants.


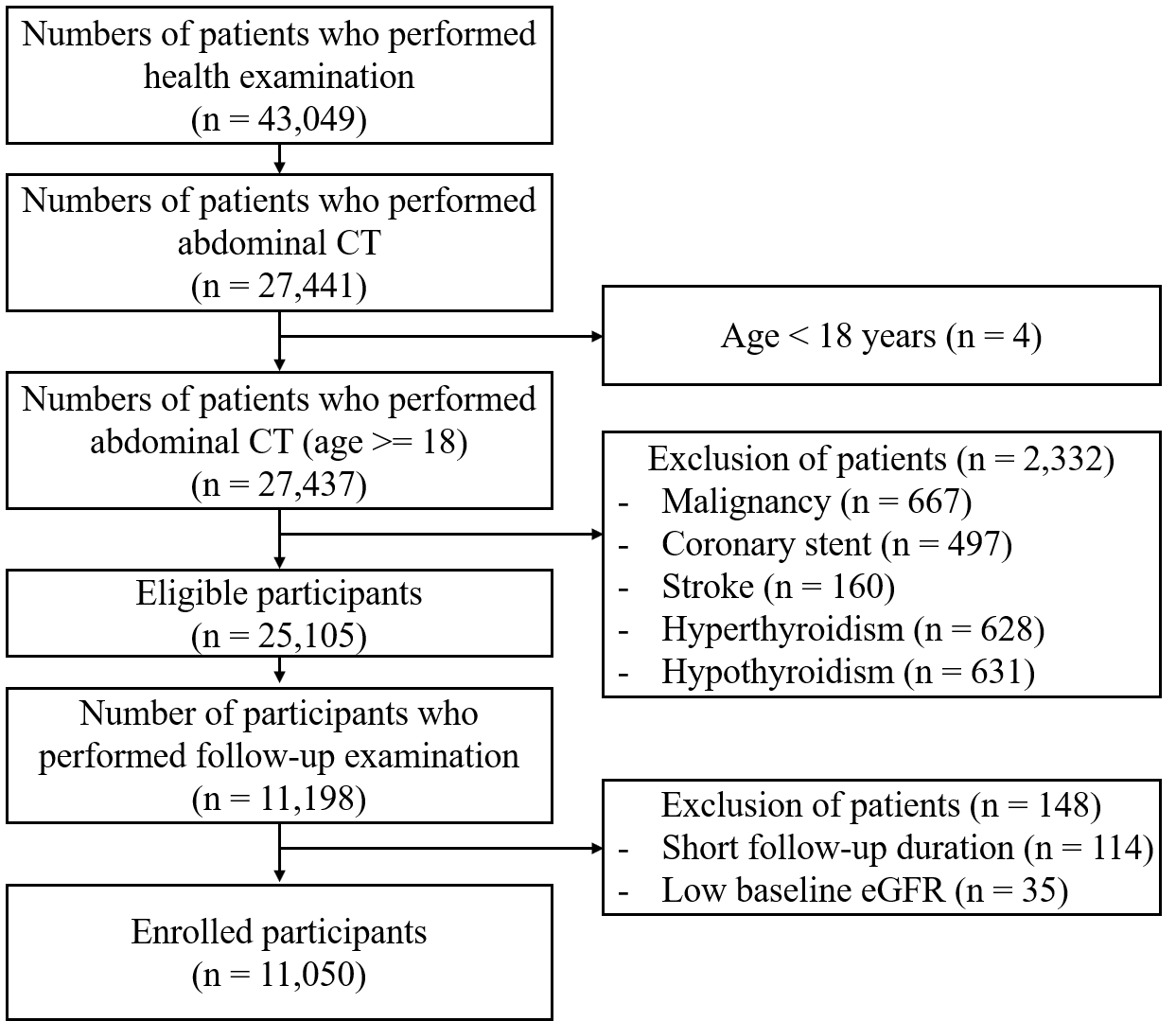


Supplementary Figure 2. Receiver operating characteristics analysis of obesity indicators for chronic kidney disease development.


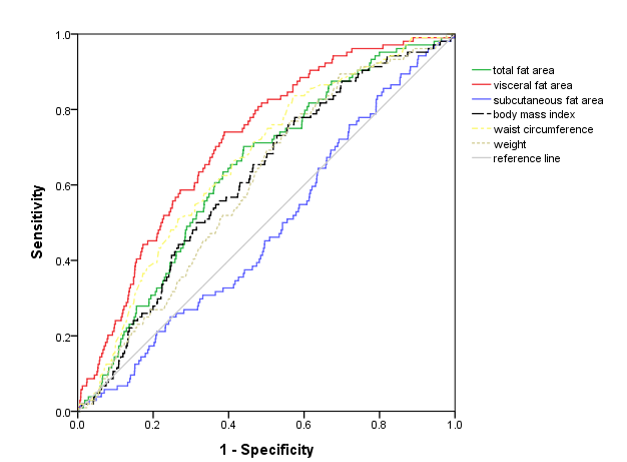


Supplementary Table 1. Characteristics of study subjects at the time of follow-up according to the development of chronic kidney disease

|  | Total participants  (N= 11,050) | CKD non-progressors  (N = 10,946) | CKD progressors  (N = 104) | *P*-value |
| --- | --- | --- | --- | --- |
| Age, years | 55.8 ± 9.2 | 55.7 ± 9.2 | 67.0 ± 9.4 | < 0.001 |
| Sex, male | 7,446 (67.4%) | 7,357 (67.2%) | 89 (85.6%) | < 0.001 |
| Diabetes mellitus | 1,446 (13.1%) | 1,416 (12.9%) | 30 (28.8%) | < 0.001 |
| Hypertension | 3,566 (32.3%) | 3,503 (32.0%) | 63 (60.6%) | < 0.001 |
| Systolic blood pressure, mmHg | 117.1 ± 13.5 | 117.0 ± 13.5 | 120.4 ± 15.5 | 0.012 |
| Diastolic blood pressure, mmHg | 77.0 ± 10.1 | 77.0 ± 10.1 | 77.1 ± 10.8 | 0.885 |
| Smoking status, (N = 10,091) |  |  |  | 0.892 |
| Never smoker | 4,309 (42.7%) | 4,258 (42.6%) | 51 (53.7%) |  |
| Current smoker | 3,673 (36.4%) | 3,639 (36.4%) | 34 (35.8%) |  |
| Ex-smoker | 2,109 (20.9%) | 2,099 (21.0%) | 10 (10.5%) |  |
| Alcohol drinking | 6,326 (57.2%) | 6,226 (57.2%) | 60 (57.7%) | 0.683 |
| Salt preference score | 0.71 ± 0.86 | 0.71 ± 0.86 | 0.43 ± 0.53 | 0.390 |
| Regular exercise | 7,539 (68.2%) | 7,465 (68.2%) | 74 (71.2%) | 0.606 |
| NSAID usage | 177 (1.6%) | 175 (1.6%) | 2 (1.9%) | 0.896 |
| Measurements of obesity |  |  |  |  |
| Weight, kg | 66.2 ± 11.2 | 66.2 ± 11.3 | 68.8 ± 9.6 | 0.007 |
| Body mass index, kg/m^2^ | 23.7 ± 2.9 | 23.7 ± 2.9 | 24.4 ± 2.6 | 0.018 |
| Waist circumference, cm | 85.4 ± 8.3 | 85.3 ± 8.3 | 89.2 ± 7.5 | < 0.001 |
| Areas of abdominal adipose tissue, (N = 1,665) |  |  |  |  |
| Total fat, cm^2^ | 275.6 ± 93.5 | 275.6 ± 93.6 | 276.0 ± 74.1 | 0.991 |
| Visceral fat, cm^2^ | 125.8 ± 56.6 | 125.6 ± 56.5 | 166.1 ± 68.0 | 0.059 |
| Subcutaneous fat, cm^2^ | 149.8 ± 56.5 | 149.9 ± 56.6 | 109.9 ± 51.5 | < 0.001 |
| Visceral vs. subcutaneous fat ratio, % | 89.8 ± 41.2 | 89.5 ± 40.9 | 150.0 ± 55.6 | < 0.001 |
| Other metabolic risk factors |  |  |  |  |
| Fasting glucose, mg/dl | 101.8 ± 19.1 | 101.8 ± 19.1 | 107.0 ± 21.5 | 0.014 |
| HbA1c, % (N = 11,002) | 5.8 ± 0.6 | 5.8 ± 0.6 | 6.0 ± 0.7 | < 0.001 |
| Uric acid, mg/dl | 5.5 ± 1.4 | 5.5 ± 1.4 | 6.6 ± 1.7 | < 0.001 |
| Cholesterol, total, mg/dl | 193.0 ± 34.3 | 193.2 ± 34.3 | 174.6 ± 36.2 | < 0.001 |
| HDL cholesterol, mg/dl | 52.8 ± 12.2 | 52.8 ± 12.2 | 49.1 ± 11.3 | 0.002 |
| LDL cholesterol, mg/dl | 117.2 ± 31.3 | 117.3 ± 31.3 | 101.0 ± 31.6 | < 0.001 |
| Triglyceride, mg/dl | 116.6 ± 75.9 | 116.6 ± 76.0 | 122.8 ± 63.9 | 0.407 |
| Serum creatinine, mg/dl | 0.87 ± 0.18 | 0.86 ± 0.17 | 1.36 ± 0.46 | < 0.001 |
| Estimated GFR, ml/min/1.73 m^2^ | 91.9 ± 12.0 | 92.3 ± 11.5 | 54.4 ± 7.05 | < 0.001 |

Results are expressed as frequencies (percentage) and mean values (standard deviation), as appropriate. CKD development was defined as GFR below 60 ml/min/1.73 m^2^ at follow-up health check-up.

Salt preference score, sum of three categories of salt preferences, i.e., preference of salt added soup at a meal, having salted food (fish or vegetables) two or more times a week, and having processed or instant food two or more times a week.

BMI, body mass index; HDL, high-density lipoprotein; LDL, low-density lipoprotein; NSAID, non-steroidal anti-inflammatory drug.

Supplementary Table 2. Events of progression to CKD according to the visceral and subcutaneous abdominal fat area

|  | N = 11,050 | Non-progression to CKD | Progression to CKD | *P*-value |
| --- | --- | --- | --- | --- |
| Visceral abdominal fat area (cm^2^) |  |  |  | < 0.001 |
| Q1 | 2,762 | 2,758 (99.9%) | 4 (0.1%) |  |
| Q2 | 2,763 | 2,748 (99.5%) | 15 (0.5% |  |
| Q3 | 2,763 | 2,734 (99.0%) | 29 (1.0%) |  |
| Q4 | 2,762 | 2,706 (98.0%) | 56 (2.0%) |  |
| Subcutaneous abdominal fat area (cm^2^) |  |  |  | 0.284 |
| Q1 | 2,762 | 2,739 (99.2%) | 23 (0.8%) |  |
| Q2 | 2,763 | 2,729 (98.8%) | 34 (1.2%) |  |
| Q3 | 2,763 | 2,742 (99.2%) | 21 (0.8%) |  |
| Q4 | 2,762 | 2,736 (99.1%) | 26 (0.9%) |  |

Supplementary Table 3. Numbers and proportion of incident chronic kidney disease and distribution of visceral adipose tissue area in subgroup populations

|  | Numbers (proportions) of incident CKD | Visceral adipose tissue area (cm^2^) |
| --- | --- | --- |
| All participants (n = 11,050) | 104 (0.9%) | 117.4 ± 54.9 |
| Ages |  |  |
| ≥ 60 years old (n = 1,616) | 62 (3.7%) | 134.7 ± 53.2 |
| < 60 years old (n = 9,372) | 42 (0.4%) | 114.3 ± 54.6 |
| Sex |  |  |
| Male (n = 7,446) | 89 (1.2%) | 135.5 ± 51.3 |
| Female (n = 3,604) | 15 (0.4%) | 80.0 ± 41.5 |
| Diabetes mellitus |  |  |
| Non-diabetes (n = 10,045) | 78 (0.8%) | 113.8 ± 53.7 |
| Diabetes mellitus (n = 1,005) | 26 (2.6%) | 153.1 ± 53.6 |
| ^*^Body mass index (n = 10,733) |  |  |
| ≥ 18.5, < 25 (n = 7,178) | 52 (0.7%) | 99.6 ± 44.7 |
| ≥ 25 (n = 3,555) | 52 (1.5%) | 160.7 ± 46.7 |
| Hypertension |  |  |
| without hypertension (n = 8,206) | 44 (0.5%) | 108.2 ± 52.6 |
| with hypertension (n = 2,844) | 60 (2.1%) | 143.9 ± 52.8 |
| Hyperuricemia |  |  |
| without hyperuricemia (n = 9,170) | 67 (0.7%) | 111.6 ± 53.5 |
| with hyperuricemia (n = 1,880) | 37 (2.0%) | 145.7 ± 52.5 |

Visceral adipose tissue areas were expressed with mean ± standard deviation.

^*^In the subgroup analysis stratified by body mass index (BMI), underweight populations (BMI < 18.5, n = 317) among the whole participants (n = 11,050) were excluded.

Supplementary Table 4. Sensitivity analysis of the risk of chronic kidney disease in relation to the visceral adipose tissue area without diabetes mellitus, hypertension, and hyperuricemia

|  |  | Univariable |  |  | Multivariable 1 |  |  | Multivariable 2 |  |
| --- | --- | --- | --- | --- | --- | --- | --- | --- | --- |
|  | N | HR (95% CI) | *P*-value | N | HR (95% CI) | *P*-value | N | HR (95% CI) | *P*-value |
| Non-diabetes mellitus  (N = 10,045) |  |  |  |  |  |  |  |  |  |
| Visceral adipose tissue area (cm^2^) |  |  | < 0.001 |  |  | 0.001 |  |  | 0.003 |
| Q1, reference | 2,511 | 1 |  | 2,508 | 1 |  | 2,430 | 1 |  |
| Q2 | 2,511 | 3.13 (1.02-9.60) | 0.046 | 2,505 | 2.83 (0.86-9.26) | 0.086 | 2,412 | 3.06 (0.90-10.43) | 0.074 |
| Q3 | 2,512 | 4.84 (1.65-14.21) | 0.004 | 2,509 | 2.21 (0.67-7.25) | 0.191 | 2,424 | 2.85 (0.82-9.86) | 0.098 |
| Q4 | 2,511 | 10.99 (3.94-30.64) | < 0.001 | 2,504 | 6.15 (1.84-20.58) | 0.003 | 2,429 | 7.13 (2.01-25.30) | 0.002 |
| No hypertension  (N = 8,206) |  |  |  |  |  |  |  |  |  |
| Visceral adipose tissue area (cm^2^) |  |  | < 0.001 |  |  | 0.005 |  |  | 0.003 |
| Q_1_, reference | 2,051 | 1 |  | 2,050 | 1 |  | 1,986 | 1 |  |
| Q_2_ | 2,052 | 1.37 (0.31-6.12) | 0.681 | 2,045 | 1.87 (0.38-9.19) | 0.443 | 1,973 | 1.69 (0.34-8.50) | 0.522 |
| Q_3_ | 2,052 | 4.47 (1.27-15.67) | 0.020 | 2,050 | 3.67 (0.87-15.51) | 0.077 | 1,978 | 3.56 (0.83-15.37) | 0.089 |
| Q_4_ | 2,051 | 8.61 (2.59-28.60) | < 0.001 | 2,047 | 8.88 (2.01-39.30) | 0.004 | 1,980 | 9.21 (2.03-41.80) | 0.004 |
| No hyperuricemia  (N = 9.170) |  |  |  |  |  |  |  |  |  |
| Visceral adipose tissue area (cm^2^) |  |  | < 0.001 |  |  | 0.003 |  |  | 0.008 |
| Q_1_, reference | 2,292 | 1 |  | 2,291 | 1 |  | 2,219 | 1 |  |
| Q_2_ | 2,293 | 3.35 (0.92-12.16) | 0.067 | 2,286 | 2.33 (0.60-9.01) | 0.219 | 2,201 | 2.14 (0.54-8.54) | 0.280 |
| Q_3_ | 2,293 | 6.04 (1.78-20.49) | 0.004 | 2,290 | 3.07 (0.82-11.55) | 0.097 | 2,205 | 3.34 (0.89-12.58) | 0.074 |
| Q_4_ | 2,292 | 12.70 (3.91-41.26) | < 0.001 | 2,287 | 6.82 (1.77-26.23) | 0.005 | 2,222 | 6.40 (1.66-24.73) | 0.007 |

N, numbers of participants; HR, hazard ratio; CI, confidence interval

Q_1_~Q_4_, quartile group of each abdominal adipose tissue area

Multivariate model 1 analysis was adjusted for age, sex, baseline eGFR, body mass index, hypertension, diabetes mellitus, smoking, dyslipidemia, and uric acid.

Multivariate model 2 analysis was adjusted for covariates in model 1 and NSAID usage, alcohol consumption, regular exercise, and salt and soda preferences.

Supplementary Table 5. Statistics of receiver operating characteristics for chronic kidney disease development and obesity indicators

|  | AUC (95% CI) | Cut-off value | Sensitivity | Specificity | *P*_1_ | *P*_2_ | *P*_3_ |
| --- | --- | --- | --- | --- | --- | --- | --- |
| Total fat area (cm^2^) | 0.64 (0.59-0.69) | 273.3 | 70.2% | 56.0% | < 0.001 | 0.27 | reference |
| Visceral fat area (cm^2^) | 0.71 (0.67-0.76) | 131.4 | 74.0% | 61.2% | < 0.001 | < 0.001 | < 0.001 |
| Subcutaneous fat area (cm^2^) | 0.52 (0.47-0.57) | 143.2 | 61.5% | 47.4% | 0.51 | 0.03 | 0.01 |
| VAT/SAT ratio (%) | 0.71 (0.67-0.76) | 114.8 | 57.7% | 77.3% | < 0.001 | 0.004 | 0.03 |
| Weight (kg) | 0.60 (0.55-0.65) | 64.6 | 76.9% | 43.8% | < 0.001 | 0.51 | 0.15 |
| Body mass index (kg/m^2^) | 0.61 (0.56-0.66) | 23.2 | 77.9% | 42.5% | < 0.001 | reference | 0.27 |
| Waist circumference (cm) | 0.66 (0.61-0.71) | 84.1 | 83.7% | 42.8% | < 0.001 | 0.003 | 0.19 |
| Weight (kg) | 0.60 (0.55-0.65) | 64.6 | 76.9% | 43.8% | < 0.001 | 0.51 | 0.15 |

*P*_1_, Wilcox test for difference with area under the curve value of 0.5.

*P*_2_, Delong test for difference with body mass index

*P*_3_, Delong test for difference with total fat area

Supplementary Table 6. Number and proportion of CKD development according to obesity defined by BMI and central obesity by visceral adipose tissue (n = 10,733)

|  | 18.5 ≤ BMI < 25 (n = 7,178) | BMI ≥ 25 (n = 3,555) |
| --- | --- | --- |
| VAT < 154.3 (n = 7,970) | 6,304, CKD 29 (0.5%) | 1,666 CKD 17 (1.0%) |
| VAT ≥ 154.3 (n = 2,763) | 874, CKD 21 (2.4%) | 1,889, CKD 102 (1.0%) |

Central obesity was defined by visceral adipose tissue 75 percentile (154.3 cm^2^).

Underweight populations (BMI < 18.5, n = 317) among the whole participants (n = 11,050) were excluded in this analysis.
