# Supplementary material for: Association of intraabdominal fat with the risk of incident chronic kidney disease according to body mass index among Korean adults
Source: PLoS One. 2023 Feb 9;18(2):e0280766. doi: 10.1371/journal.pone.0280766 (PMC9910748; doi:10.1371/journal.pone.0280766)
Supplement: Attachment — Submitted filename: Response to Reviewers (rebuttal letter) 0831.docx [file pone.0280766.s002.docx]

**Comments from the Editor and Reviewers**

Editor comments
Thank you for submitting your manuscript to PLOS ONE. After careful consideration, we feel that it has merit but does not fully meet PLOS ONE’s publication criteria as it currently stands. Therefore, we invite you to submit a revised version of the manuscript that addresses the points raised during the review process.

Please submit your revised manuscript by September 1, 2022. If you will need more time than this to complete your revisions, please reply to this message or contact the journal office at plosone@plos.org. When you're ready to submit your revision, log on to https://www.editorialmanager.com/pone/ and select the 'Submissions Needing Revision' folder to locate your manuscript file.

Please include the following items when submitting your revised manuscript:

1) A rebuttal letter that responds to each point raised by the academic editor and reviewer(s). You should upload this letter as a separate file labeled 'Response to Reviewers'.
2) A marked-up copy of your manuscript that highlights changes made to the original version. You should upload this as a separate file labeled 'Revised Manuscript with Track Changes'.
3) An unmarked version of your revised paper without tracked changes. You should upload this as a separate file labeled 'Manuscript'.

If you would like to make changes to your financial disclosure, please include your updated statement in your cover letter. Guidelines for resubmitting your figure files are available below the reviewer comments at the end of this letter.

If applicable, we recommend that you deposit your laboratory protocols in protocols.io to enhance the reproducibility of your results. Protocols.io assigns your protocol its own identifier (DOI) so that it can be cited independently in the future. For instructions see: <https://journals.plos.org/plosone/s/submission-guidelines#loc-laboratory-protocols>. Additionally, PLOS ONE offers an option for publishing peer-reviewed Lab Protocol articles, which describe protocols hosted on protocols.io. Read more information on sharing protocols at https://plos.org/protocols?utm_medium=editorial-email&utm_source=authorletters&utm_campaign=protocols..
**Response:** On behalf of all coauthors, I would like to thank the editor and anonymous reviewers for the critical comments on this manuscript. We have addressed all comments and revised the manuscript accordingly. Please refer to our changes and detailed explanations regarding the reviewers’ comments in the revised manuscript and rebuttal letter. All changes are shown in blue font both below and in the revised manuscript.

Reviewer Comments:

Reviewer 1
The manuscript addresses the study of CKD compared to the BMI of exclusively Korean individuals using CT. In general, the data showed that individuals with a normal BMI, but with visceral adipose tissue (VAT) had a higher risk of CKD than individuals with a higher BMI associated with a lower muscle mass. Other studies have already shown this contradictory effect in which obese individuals have a lower risk of CKD, however without assessing VAT by means of CT. This study corroborates other findings in this context and reinforces the importance of evaluating the TAV with greater precision, for example, through CT, and thus establishing clinical methods to monitor as well as reduce the formation of TAV.

**Response:** We appreciate the reviewer’s valuable comments and positive evaluation of our revisions.

1 – In the full title, must correct the word visceral

**Response:** We appreciate the reviewer's valuable comments. Following the reviewer’s comment, we fixed the word "visceral adipose tissue" to the intraabdominal fat.

2 – Initials NSAID in the line 172 (Statistical analysis) must be defined.

**Response:** Thank you for valuable comment. We added the have full name of NSAID abbreviation as follows: The multivariable model used age, sex, baseline eGFR, BMI, hypertension, diabetes mellitus, smoking, dyslipidemia, uric acid, NSAID (non-steroidal anti-inflammatory drug) usage, alcohol consumption, regular exercise, and salt and soda preferences as covariates.

3 – To better understanding - lines 208, 209 “…VAT (P < 0.001, Supplementary Table 1 and Fig. 1A). However, SAT was not associated with CKD progression (Fig. 1B).”

**Response:** We appreciate the reviewer's important comment. According to the reviewer’s comment, the following details were provided regarding existing sentences to help readers better comprehend them: “Numbers and probability of CKD progression increased significantly with an increase in VAT (*P* < 0.001, S1 Table and Fig 1). The risk of CKD progression increased according to the increase of VAT categorized into quartiles (*P* < 0.001, S1 Table). In Kaplan-Meier analysis, the probability of CKD progression increased according to the increase of VAT (*P* < 0.001, Fig. 1). However, an increase in SAT was not associated with CKD progression (*P* = 0.284, S1 Table; *P* = 0.209, Fig. 1).”

4 - Lines 263-267 the authors report ”Olivo et al. investigated 1,477 African Americans with a majority of females (63%) and followed up for a median 8 (6-11) years with 12.4% CKD incidence. They reported that VAT was not associated with incident CKD [26].” In fact, according Olivo et al “BMI and waist circumference z-score were not associated with incident CKD when modeled continuously (P=0.1 for each). However, higher visceral adipose volume was associated with an increased risk of incident CKD overall (P=0.008), but was nonlinear (Figure 1; P=0.02).” By this reason, the authors must review their discussion at this point.

**Response:** Thank you for valuable comment. Olivo et al. summarized and concluded their investigation as “we observed an association between higher visceral adiposity and an increased risk for new-onset CKD in African American adults that was only present among those with poor diet quality”. However, as the reviewer pointed out, we agree that the way of description on previous research of Olive et al. had chance of misunderstanding. Therefore, we added detailed explanations on previous investigation as follows: “VAT categorized into quartiles was not associated with incident CKD and the risk for CKD according to VAT (in continuous analysis model) showed U-shaped nonlinear relationship [26]. Effects of VAT on incident CKD had significant interaction with dietary quality and VAT was associated with CKD development only in the low dietary quality group.”

5 – Lines 270-271 - “In the study of Olivo et al., VAT might be associated with an insignificant risk of incident CKD due to the relatively low numbers of participants [26]”. However, in the study by Olivo et al there is the information "The key strength of our investigation was the use of a large, prospective, high-risk study population with long-term follow-up." Thus, there is a contradiction between the manuscript and Olivo's study, which implies the need to review the discussion by the authors.

**Response:** We appreciate the reviewer' important comment. Following the comment of the reviewer, we changed the discussion in our manuscript so that it doesn't contradict what Olivo et al. described in their study. “Discrepancies with our studies’ results could be caused by the differences in enrolled participants’ ethnicity, gender distribution, dietary quality, and metabolic profiles”. “Our study’s participant showed male preponderance. In general, Asian population have low prevalence of obesity and metabolic risk for CKD compared to the African Americans [27]. In addition, sexual difference is an important determinant for obesity-related CKD development [28, 29]. Moreover, it has been reported that there is a difference in food intake pattern and quality between different ethnicities. Asian groups scored highly for diet quality due to higher fish intakes and lower fat intakes compared to other groups, whereas African American groups were noted to be among the least fruit and vegetable eaters [30]. These differences in dietary pattern according to ethnicity can be an important determinant for the CKD development [31, 32].

27. Sabanayagam C, Lim SC, Wong TY, Lee J, Shankar A, Tai ES (2010) Ethnic disparities in prevalence and impact of risk factors of chronic kidney disease. Nephrol Dial Transplant 25: 2564-2570.<https://doi.org/10.1093/ndt/gfq084>

28. Chen IJ, Hsu LT, Lu MC, Chen YJ, Tsou MT, Chen JY (2021) Gender Differences in the Association Between Obesity Indices and Chronic Kidney Disease Among Middle-Aged and Elderly Taiwanese Population: A Community-Based Cross-Sectional Study. Front Endocrinol (Lausanne) 12: 737586.<https://doi.org/10.3389/fendo.2021.737586>

29. Sakurai M, Kobayashi J, Takeda Y, Nagasawa SY, Yamakawa J, Moriya J, et al. (2016) Sex Differences in Associations Among Obesity, Metabolic Abnormalities, and Chronic Kidney Disease in Japanese Men and Women. J Epidemiol 26: 440-446.<https://doi.org/10.2188/jea.JE20150208>

30. Bennett G, Bardon LA, Gibney ER (2022) A Comparison of Dietary Patterns and Factors Influencing Food Choice among Ethnic Groups Living in One Locality: A Systematic Review. Nutrients 14.<https://doi.org/10.3390/nu14050941>

31. Asghari G, Momenan M, Yuzbashian E, Mirmiran P, Azizi F (2018) Dietary pattern and incidence of chronic kidney disease among adults: a population-based study. Nutr Metab (Lond) 15: 88.<https://doi.org/10.1186/s12986-018-0322-7>

32. Bach KE, Kelly JT, Palmer SC, Khalesi S, Strippoli GFM, Campbell KL (2019) Healthy Dietary Patterns and Incidence of CKD: A Meta-Analysis of Cohort Studies. Clin J Am Soc Nephrol 14: 1441-1449.<https://doi.org/10.2215/CJN.00530119>
