# Supplementary material for: Association of intraabdominal fat with the risk of incident chronic kidney disease according to body mass index among Korean adults
Source: PLoS One. 2023 Feb 9;18(2):e0280766. doi: 10.1371/journal.pone.0280766 (PMC9910748; doi:10.1371/journal.pone.0280766)
Supplement: Attachment — Submitted filename: Response to Reviewers (rebuttal letter)1210.docx [file pone.0280766.s003.docx]

**Comments from the Editor and Reviewers**

Editor comments
Thank you for submitting your manuscript to PLOS ONE. After careful consideration, we feel that it has merit but does not fully meet PLOS ONE’s publication criteria as it currently stands. Therefore, we invite you to submit a revised version of the manuscript that addresses the points raised during the review process.

Please submit your revised manuscript by Dec 10 2022 11:59PM. If you will need more time than this to complete your revisions, please reply to this message or contact the journal office at plosone@plos.org. When you're ready to submit your revision, log on to https://www.editorialmanager.com/pone/ and select the 'Submissions Needing Revision' folder to locate your manuscript file.

Please include the following items when submitting your revised manuscript:

- A rebuttal letter that responds to each point raised by the academic editor and reviewer(s). You should upload this letter as a separate file labeled 'Response to Reviewers'.
- A marked-up copy of your manuscript that highlights changes made to the original version. You should upload this as a separate file labeled 'Revised Manuscript with Track Changes'.
- An unmarked version of your revised paper without tracked changes. You should upload this as a separate file labeled 'Manuscript'.

If you would like to make changes to your financial disclosure, please include your updated statement in your cover letter. Guidelines for resubmitting your figure files are available below the reviewer comments at the end of this letter.

If applicable, we recommend that you deposit your laboratory protocols in protocols.io to enhance the reproducibility of your results. Protocols.io assigns your protocol its own identifier (DOI) so that it can be cited independently in the future. For instructions see: https://journals.plos.org/plosone/s/submission-guidelines#loc-laboratory-protocols. Additionally, PLOS ONE offers an option for publishing peer-reviewed Lab Protocol articles, which describe protocols hosted on protocols.io. Read more information on sharing protocols at https://plos.org/protocols?utm_medium=editorial-email&utm_source=authorletters&utm_campaign=protocols.

**Response:** On behalf of all coauthors, I would like to thank the editor and anonymous reviewers for the critical comments on this manuscript. We have addressed all comments and revised the manuscript accordingly. Please refer to our changes and detailed explanations regarding the reviewers’ comments in the revised manuscript and rebuttal letter. All changes are shown in blue font both below and in the revised manuscript.

Reviewer Comments:

Reviewer #2.
Recently, central obesity affects metabolic syndrome, albuminuria and worse outcomes in the kidney. Present study well demonstrated visceral adiposity is associated with incident CKD in general population. Lee et al evaluated visceral adiposity using abdominal fat CT. More accurate evaluation for central obesity is a strength in this study.

**Response:** We appreciate the reviewer’s valuable comments and positive evaluation of our revisions.

Reviewer #3.
The authors evaluated the association of intraabdominal fat (measured by computed tomography) with the risk of chronic kidney disease (CKD) in a large Korean population. This study found that visceral adipose tissue (VAT) instead of subcutaneous abdominal fat was associated with incident CKD, especially among participants with normal weight and central obesity. The message was straightforward.

However, the major concern is that VAT has been a known risk factor for diabetes, hypertension, and hyperuricemia. These three diseases are all major contributors to the development of CKD. It is easy to be concerned that VAT is not an independent risk factor for CKD, although adjustment for diabetes, blood pressure, and uric acid levels. To address this issue, I am curious about whether the authors could do the below analyses if possible.

1. Stratified analyses by diabetes (has been done), hypertension, and hyperuricemia.

**Response:** We appreciate the reviewer’s valuable comments and positive evaluation of our revisions. Subgroup analysis results stratified by hypertension and hyperuricemia was added to Fig 2. Risk for incident CKD was prominently discriminated according to age, sex, and body mass index. However, we could not find apparent difference in the risk for incident CKD according to the diabetes mellitus, hypertension, and hyperuricemia in multivariable analysis model. We added the results in the Result section as follows: “The risk of CKD by VAT in each subgroup was compared (Fig 2 and S3 Table). Adjusted HRs for CKD progression was comparable according to diabetes mellitus, hypertension, and hyperuricemia. However, risk for CKD development were more prominent in participants with lower age (<60 years old), female, and normal-weight (18.5 ≤ BMI < 25) group.”

2. Sensitivity analysis excluding all the participants with diabetes, hypertension, and hyperuricemia.

**Response:** Thank you for valuable comment. To clarify the results of this study, we additionally performed sensitivity analysis excluding all the participants with diabetes, hypertension, and hyperuricemia. The results of this sensitivity analysis are added in S4 Table. VAT was significantly associated with incident CKD in all sensitivity analysis model. We added this result in the Result section as follows: “Sensitivity analysis was performed excluding participants with diabetes mellitus, hypertension, and hyperuricemia (S4 Table). Increased VAT was consistently associated with increased risk for incident CKD in all sensitivity analysis model.”

3. Mediation analyses to explore the mediation effects of these three diseases on the association between VAT and CKD.

**Response:** We appreciate the reviewer's important comment. Central obesity is a key component of metabolic syndrome and well-known risk factor for insulin-resistance. Consequently, it is plausible that comorbidities such as diabetes, hypertension, and hyperuricemia mediated the effects of VAT on incident CKD. Following the valuable comments of the reviewer, we performed mediation analysis using the R Medflex package software (Steen J, Loeys T, Moerkerke B, Vansteelandt S. Medflex: an R package for flexible mediation analysis using natural effect models. J Stat Softw 2017. doi: <https://doi.org/10.18637/jss.v076.i11>.)

| **Mediation analysis** | **Mediator** | | | |
| --- | --- | --- | --- | --- |
|  | **Diabetes mellitus HR (95% CI)** | **Hypertension**  **HR (95% CI)** | **Hyperuricemia HR (95% CI)** | **Uric acid HR (95% CI)** |
| Indirect effect | 1.053  (0.893, 1.233) | 1.056  (0.891, 1.243) | 1.028  (0.869, 1.212) | 1.024  (0.861, 1.210) |
| Direct effect | 2.355  (2.104, 2.630) | 2.434  (2.166, 2.716) | 2.438  (1.179, 2.744) | 2.427  (2.160, 2.714) |
| Total effect | 2.480  (2.068, 2.968) | 2.572  (2.142, 3.083) | 2.506  (2.095, 3.021) | 2.486  (2.071, 2.983) |

As shown in the table above, none of the indirect effects of diabetes, hypertension, hyperuricemia, and uric acid were confirmed to be significant. However, direct effect of VAT was consistently associated with increased risk for incident CKD in all mediation analysis model. There are several possible explanations for why the mediating effects of diabetes, hypertension, and hyperuricemia were not validated in this study's analytical results. First, a follow-up period of 5.6 years is relatively short. Generally, it is known that diabetes mellitus can lead to CKD in 5 to 10 years or more. In addition, as noted in the discussion section, the enrolled participants are all Asian and the prevalence of diabetes mellitus and hypertension are low compared to the other studies (Madero et al, doi.org/10.2215/CJN.07010716 and Olivo et al. doi.org/10.1093/ndt/gfx230). In this study, multivariate analysis including hypertension, diabetes mellitus, and hyperuricemia was conducted to adjust the effects from these comorbidities. Longitudinal prospective follow-up research with a larger sample size and longer duration is required to confirm the mediation effect of these comorbidities.

4. Provide the participants’ characteristics shown in table 1 by CKD at the follow-up health examination.

**Response:** Thank you for valuable comment. We added the descriptive statistics for the study subjects at the time of follow-up in the Supplementary Table 1. We added this result in the Result section as follows: “At the time of enrollment, no patients had decreased eGFR (<60 ml/min/1.73m^2^). Participants were followed up for 5.6 ± 2.6 (range 1.0-11.9) years (S1 Table). Follow-up serum creatinine levels were 0.87 ± 0.18 mg/dl and eGFR was 91.9 ± 12.0 ml/min/1.73m^2^. During the follow-up period, 104 (0.94%) participants progressed to CKD. Overall incidence rate of CKD was 1.69 cases per 1,000 person-years. Progressors to CKD showed higher mean age (61.1 ± 9.0 vs. 50.1 ± 8.8, *P*< 0.001) and male preponderance (85.6% vs. 67.2%, *P*< 0.001) than non-progressors. Prevalence of diabetes mellitus and hypertension was higher among progressors to CKD than among non-progressors. Progressors to CKD showed higher baseline TAT and VAT than non-progressors. However, SAT was comparable between the groups.”
